# Uganda Public Health Fellowship Program's Contributions to the National HIV and TB Programs, 2015–2020

**DOI:** 10.9745/GHSP-D-21-00574

**Published:** 2022-04-28

**Authors:** Alex R. Ario, Lilian Bulage, Yvette Wibabara, Peter Muwereza, Daniel Eurien, Steven N. Kabwama, Benon Kwesiga, Daniel Kadobera, Stavia Turyahabwe, Joshua B. Musinguzi, Rhoda K. Wanyenze, Pamela M. Nasirumbi, Deus Lukoye, Julie R. Harris, Lisa A. Mills, Lisa J. Nelson

**Affiliations:** a Ministry of Health, Kampala, Uganda.; b Uganda Public Health Fellowship Program, Ministry of Health, Kampala, Uganda.; c Uganda National Institute of Public Health, Kampala, Uganda.; d African Field Epidemiology Network, Kampala, Uganda.; e College of Health Sciences, Makerere University School of Public Health, Kampala, Uganda.; f National TB and Leprosy Control Division, Ministry of Health, Kampala, Uganda.; g AIDS Control Programme, Ministry of Health, Kampala, Uganda.; h U.S. Centers for Disease Control and Prevention, Division of Global Health Protection, Centers for Global Health, Kampala, Uganda.; i U.S. Centers for Disease Control and Prevention, Division of Global HIV and TB, Centers for Global Health, Kampala, Uganda.

## Abstract

Despite remarkable progress in controlling HIV and TB, Uganda is one of the 30 high-burden TB/HIV countries. Approximately 53,000 Ugandans had a new HIV diagnosis in 2019, and approximately 88,000 Ugandans had a TB diagnosis in 2020. Fellows in the Uganda Public Health Fellowship Program (UPHFP) work directly with the Ministry of Health AIDS and TB Control Programs, the U.S. Centers for Disease Control and Prevention, UPHFP supervisors, and implementing partners to investigate and evaluate HIV-related and TB-related issues. These activities have contributed to the Uganda HIV and TB programs. UPHFP fellows complete projects in 7 competency domains, including outbreak investigations, surveillance evaluations, and data quality improvement. Priority HIV/AIDS/TB information gaps/topics are identified in consultation with key stakeholders, and fellows complete projects to guide program improvements and policy decisions. During 2015–2020, UPHFP fellows implemented 127 HIV and TB projects covering key program areas in AIDS and TB control programs, including care and treatment (16 projects), TB/HIV (18), prevention of mother-to-child HIV transmission (24), key and priority populations (9), pre-exposure and post-exposure prophylaxis (7), adolescent girls and young women (6), service delivery (13), and diagnosis of TB including drug-resistant TB and TB in high-risk groups (32). These projects have helped improve retention, quality of care, and treatment outcomes for people living with HIV, HIV and TB coinfected patients, and TB patients. They have also contributed to the decrease in pediatric TB and infant HIV positivity rates and improved service delivery for key populations. UPHFP results were disseminated to relevant stakeholders such as government departments, implementing partners, districts, and the general community and guided decision making. UPHFP has significantly improved HIV and TB control in Uganda. Other countries with similar programs could benefit from this approach and utilize program fellows to support HIV and TB control.

## Abstract (precis)

The Uganda Public Health Fellowship Program has built the capacity of its fellows to address multiple gaps in the Uganda health system as well as to contribute to improving Uganda's ability to prevent, prepare for, and respond to public health emergencies such as HIV and TB.

## Teaser

**Key Findings:**

- During 2015–2020, the Uganda Public Health Fellowship Program (UPHFP) a total of 67 fellows have been trained and have worked on 127 HIV and TB-related epidemiology projects.
- Involving UPHFP fellows in timely, programmatically relevant projects has increased effectiveness in HIV and TB prevention and control, and integrating UPHFP within the Ministry of Health has enabled Uganda to move steadily toward achieving HIV and TB epidemic control.

## INTRODUCTION

Uganda has made tremendous progress in controlling HIV, reducing infections from a national prevalence of 30% in 1992 to 6.2% in 2020 and halving the number of annual new HIV infections from 94,000 in 2010 to 53,000 in 2019.^1^ Approximately 1.34 million of Uganda's 37 million people (projected from the 2014 census) are HIV positive. The 2016/2017 population-based Uganda HIV/AIDS Impact Assessment survey showed a 6.2% HIV prevalence among adults aged 15–64 years (women, 7.6%; men, 4.7%), compared with 7.3% in 2011.^2^

Despite economic constraints and major public health challenges,^3^ Uganda has implemented programs providing antiretroviral therapy (ART); safe voluntary medical male circumcision; prevention of mother-to-child HIV transmission interventions; pre-exposure and post-exposure prophylaxis; and TB control, diagnosis, and treatment.^4^ Well-designed policies, strong leadership by government and civil society organizations, support from donors and implementing partners, and human resource capacity-building programs, such as the Uganda Public Health Fellowship Program (UPHFP), have enabled the implementation and evaluation of these programs.^5^

UPHFP is an in-service, post-master's-degree field epidemiology training program established in 2015 by the Uganda Ministry of Health (MOH), with the support of key partners including Makerere University School of Public Health (which manages the project funds and provides lecturers to teach fellows) and the U.S. Centers for Disease Control and Prevention (CDC) (which funds the program and also provides technical assistance through a resident advisor who is an epidemiologist). UPHFP is part of the Uganda National Institute of Public Health, an integrated disease control center that provides evidence-based leadership and public health services, which helps catalyze responses of the country to important public health challenges, including global health security. The institute is science-based, and hence a trusted source of information and evidence for policy makers and decision makers. We describe UPHFP's contribution to the country's HIV and TB programs during 2015–2020 to demonstrate how a program such as UPHFP can be designed to address public health needs.

## UGANDA PUBLIC HEALTH FELLOWSHIP PROGRAM IMPLEMENTATION, 2015–2020

UPHFP is a 2-year, nondegree-granting, full-time, competency-based fellowship program modeled after the U.S. Epidemic Intelligence Service program. UPHFP is designed to train mid-career health professionals in public health leadership. To qualify, candidates must have a master's degree in a health-related discipline such as public health, veterinary public health, epidemiology, nutrition, or similar fields.

During the 2-year fellowship period, UPHFP fellows are required to attain competency in 7 domains by completing a portfolio of projects in each domain. Some of these domains include but are not limited to response to public health emergencies, conducting an epidemiological study, public health surveillance data analysis and evaluation, scientific communication, and leadership and management.^6^ Fellows are placed at host sites for field project-based, hands-on training. The host sites are determined based on MOH priorities, availability of learning opportunities, the fellow's career path and interests, availability of qualified and interested host site mentors and supervisors, and accessibility of public health surveillance data. Fellows work on priority projects under the close mentorship of experienced public health professionals.^6^ Each mentor works with 1 fellow. Each fellow must complete at least 1 HIV-focused and/or TB-focused project, irrespective of their host institution. A subset of fellows is assigned to the AIDS Control Program (ACP) and the National TB and Leprosy Control Program (NTLP), and these fellows also complete more in-depth HIV- and TB-focused projects.

During the 2-year fellowship period, UPHFP fellows are required to attain competency in 7 domains by completing a portfolio of projects in each domain.

Implementation of UPHFP has been relatively smooth with a few challenges. The program's placement within the MOH and having fellows' mentors at host sites raised the demand for additional pay. In addition, fellows were viewed as students, yet they are qualified to work as officers within the MOH programs because they hold master's degrees. More so, fellows as frontline responders were required to go to the field with their counterparts in the MOH, yet the program had a caveat on funding nonprogram personnel, particularly those on the government payroll. We discussed these issues in meetings with the steering committee and top management of the MOH, and a resolution was made clarifying that the program is the MOH's initiative and the program operates within the rules of CDC funding. The mentors were told the value of hosting fellows, who are actually not students, and how to benefit from the knowledge the fellows brought. This brought a positive outlook to the program and greatly improved access to data by fellows.

The program has demonstrated its capability in contributing to effective detection, prevention, preparedness, and response to public health emergencies in the country, as exemplified by winning 2 CDC Director's awards for excellence in public health. This award is presented in recognition of significant contributions toward successful responses to public health emergencies (natural and manmade disasters and disease outbreaks.) Details about UPHFP have been described elsewhere.^5^^–^^7^

### Approaches to Achieving the HIV- and TB-Specific Deliverables for UPHFP Fellows

Early in the program, UPHFP fellows attend a comprehensive HIV and TB lecture given by subject matter experts from CDC, MOH, and Makerere University College of Health Sciences. The lecture familiarizes them with national HIV and TB epidemiology, program components and goals, and data sources. For those that need further understanding of the concepts, fellows arrange meetings with subject matter experts from CDC when needed. UPHFP staff work throughout the year with key players in HIV/AIDS and TB prevention, including MOH programs (e.g., ACP, NTLP, and Central Public Health Laboratories [CPHL]), CDC staff working to implement U.S. President's Emergency Plan for AIDS Relief (PEPFAR) goals, and implementing partners to identify key information gaps/topics on HIV and TB. A list of topics is then generated from which the fellows are assigned to HIV and TB projects. The fellows' projects are often based on existing program and survey data that require analysis, while a few others require new data collection.

For each UPHFP cohort, potential projects are collaboratively ranked based on need, practicality, and ability to generate evidence that guides program improvement, innovation, and policy decisions. Data sources used for HIV and TB projects include surveillance databases, the MOH- and CDC-approved research project datasets, and program data from ACP, NTLP, CPHL, and implementing partners. Site-level data from health facilities are also used, depending on the focus and scope of the project; some projects are undertaken at a facility or regional level in response to requests from partner facility staff and/or regional authorities such as Uganda Prisons Services, Rakai Health Sciences Program (RHSP), and Baylor Uganda. Once projects are selected and assigned, fellows generate study proposals and submit them for ethical and administrative review. MOH subject matter experts mentor the fellows who are attached to other programs within the MOH other than ACP or NTLP and provide guidance for HIV and TB-related projects when appropriate. Subsequently, fellow-mentor pairs develop a roadmap for implementing projects within an appropriate time frame (approximately 6 months).

In terms of site selection, placement of fellows is prioritized in host sites involved in PEPFAR-supported HIV and TB programs, including ACP, NTLP, CPHL, Mildmay Uganda, and RHSP. Some of these are government programs (e.g., ACP, NTLP, and CPHL), while Mildmay and RHSP are PEPFAR-supported nongovernmental organizations. At these host institutions, fellows focus on projects such as HIV survey data analysis, epidemiological investigations, and evaluation of HIV and TB service delivery programs. In 2018, an additional TB operational research course was offered to and completed by all fellows; numerous priority TB-related operational research projects were completed as a result of the course. HIV and TB projects complement other non-HIV projects undertaken by UPHFP fellows, comprising a diverse portfolio of disease-related activities including the projects done under public health emergency response, epidemiological, and cost analysis studies. All findings are disseminated in various forums in Uganda and beyond, including MOH program meetings, community and facility meetings, stakeholder meetings, conferences, PEPFAR Uganda Science Summits, epidemiological and program bulletins, and peer-reviewed journals.

### UPHFP Contributions to Uganda HIV and TB Programs

UPHFP was launched in January 2015 with 10 fellows in the first cohort. By 2020, 6 cohorts totaling 67 fellows had been recruited and trained. Over the past 6 years, fellows completed 133 applied epidemiology projects, of which 127 represented HIV/AIDS and/or TB-specific issues (Table 1 and Table 2). Fellows have examined programmatic issues and risk factors related to HIV/AIDS services uptake gaps among young people, virologic nonsuppression, quality of care in HIV service delivery, HIV/AIDS care services for key and priority population groups, knowledge and behavior related to HIV among fishing communities (a priority population for HIV response in Uganda), utilization of and quality of reporting for prevention of mother-to-child HIV transmission services, and HIV services in refugee populations. These projects have provided valuable epidemiologic evidence to guide programming in Uganda. For example, fellows' study on factors associated with virological nonsuppression among HIV-positive patients receiving ART and investigation of a multidrug-resistant TB outbreak led to new strategies to improve retention and adherence to HIV and TB treatment in Uganda (Table 3).^8^^–^^11^ These projects have provided valuable evidence and information for planning and implementation at national, regional, and district levels (Table 1 and Table 2).

**TABLE 1.** TB and HIV Projects Completed by the Uganda Public Health Fellowship Program, 2015–2020

Year	HIV	TB	TB/HIV	Leprosy^a^	Total
2015	6	0	3	0	9
2016	20	3	1	0	24
2017	15	11	6	1	33
2018	8	11	6	0	25
2019	12	6	1	1	20
2020	14	1	1	0	16
Total	75	32	18	2	127

a Captured here, a neglected tropical disease under the National TB and Leprosy Control Program.

**TABLE 2.** HIV and TB Projects Implemented by Program Area by the Uganda Public Health Fellowship Program, 2015–2020

Key Program Area	No. of Projects (N=127)
HIV care and treatment	16
Prevention of mother-to-child of HIV transmission	24
TB/HIV^a^	18
TB^a^	32
Leprosy^b^	2
Key and priority populations	9
Pre-exposure prophylaxis/post-exposure prophylaxis	7
Adolescent girls and young women	6
Service delivery	13

a Including TB operations research course projects.

b Captured here, a neglected tropical disease under the National TB and Leprosy Control Program.

**TABLE 3.** Published HIV and TB Projects Implemented by the Uganda Public Health Fellowship Program, 2015–2020^10^^–^^24^

Project Title	Year of Study or Investigation	Disease Type
Preference and uptake of different community-based HIV testing service delivery models among female sex workers along Malaba-Kampala highway, Uganda, 2017	2015	HIV
HIV prevalence and uptake of HIV/AIDS services among youths 15–24 years in fishing and surrounding communities of Kasensero, Rakai District, South Western Uganda	2015	HIV
Factors affecting quality of care for virologically non-suppressed HIV positive patients in Jinja, Buikwe and Iganga Districts	2015	HIV
Factors associated with virological non-suppression among HIV-positive patients on antiretroviral therapy in Uganda, August 2014–July 2015	2015	HIV
Evaluation of community-based HIV service delivery models for sex workers along Malaba, Kampala Highway, Uganda, 2016	2016	HIV
The burden of HIV/AIDS and uptake of services among adolescents/youth in Kasensero fishing community, Rakai District	2016	HIV
Factors associated with utilisation of couple HIV counselling and testing among HIV-positive adults in Kyoga fishing community Uganda, May 2017: cross sectional study	2016	HIV
Utilization of elimination of mother-to-child transmission services: Kampala, Uganda, 2016	2016	HIV
Comprehensive knowledge of HIV transmission among fishing villages of Lake Kyoga	2016	HIV
Risk of HIV positivity in exposed infants associated with different interventions, Uganda, 2016	2016	HIV
Tracking missed ANC appointments using Option B+ weekly SMS reporting in Central Uganda, January–June 2016	2016	HIV
Low proportion of women who came knowing their HIV status at first antenatal care visit, Uganda, 2012–2016: a descriptive analysis of surveillance data	2017	HIV
Multidrug-resistant tuberculosis outbreak associated with poor treatment adherence and delayed treatment: Arua District, Uganda, 2013–2017	2017	TB
Spatial distribution and temporal trends of leprosy in Uganda, 2012–2016: a retrospective analysis of public health surveillance data	2017	Leprosy
The yield of HIV testing during pregnancy and post-natal period in Uganda 2015–2018, a descriptive analysis of HMIS data	2019	HIV
Epidemiological profile of rifampicin-resistant TB patients in Uganda 2014–2018: analysis of laboratory data	2019	TB

A fellows' investigation of a multidrug-resistant TB outbreak led to new strategies to improve retention and adherence to TB treatment in Uganda.

Another project that improved programming was a comparison of the 90-90-90 HIV care cascade in prisons. The Joint United Nations Programme on HIV/AIDS established 90-90-90 targets to reach by 2020: 90% of individuals with HIV are aware of their HIV status; of these, 90% are receiving ART; and of these, 90% have viral load suppression. In this project, direct on-site HIV care was compared with off-site care to identify optimal approaches to achieving the 90-90-90 targets in Ugandan prisons. Results showed that on-site care was superior at achieving each of the cascade steps. Following this project, the Uganda Prisons Services' PEPFAR-funded HIV program service delivery model was modified, expanding direct government-to-government funding to establish accredited ART centers at more than 50 large-volume Uganda prisons to provide direct HIV/TB care and treatment services for more than 200 prisons nationwide.^9^ Another study focusing on community service delivery models for female sex workers provided evidence that improved service delivery to this key population.^12^

## DISSEMINATION OF FINDINGS

From 2015 to 2020, UPHFP fellows submitted 122 manuscripts to peer-reviewed scientific journals (Table 3 and Supplement); of these manuscripts, 28 focused on HIV and TB. As of December 31, 2020, 67 manuscripts have been published (HIV and TB articles^10^^–^^24^), 9 manuscripts have been accepted pending minor revisions, and 46 were in various stages of journal review. In addition to these manuscripts, UPHFP fellows have had 240 abstracts accepted for presentation at national and international conferences; of these abstracts, 50 focused on HIV and TB-related topics. Annually, the program holds a National Field Epidemiology Conference at which findings are shared with stakeholders. UPHFP fellows also have presented their work at International AIDS Society conferences, the most selective and comprehensive conference in the global HIV field. Fellows also present in the National Quality Improvement Conference, Uganda Society for Social Scientists Conference, and the Joint Annual Scientific Conference, all of which are held annually in the country.

UPHFP fellows also have presented their work in multiple national forums, including the PEPFAR Uganda Science Summit (January 2020), and in influential settings where policy and programs were being formulated, including for the annual PEPFAR Country Operating Plan (COP). The COP outlines HIV and TB priorities to be implemented in the period. UPHFP also has supported the MOH in reviving the National TB and Leprosy Program Bulletin, where the fellows and MOH epidemiologists publish important public health information. Results from UPHFP HIV and TB projects were also disseminated to relevant stakeholders, including line ministries, implementing partners, development partners, districts, and communities in Uganda. Periodically, specific program-directed meetings, especially with ACP and NTLP, review key UPHFP findings with programmatic implications. The numerous epidemiologic bulletins of the MOH (www.health.go.ug; www.uniph.go.ug) are other channels to reach decision makers in a timely fashion with evidence generated from UPHFP projects.

### Career Paths of UPHFP Graduates

After completing the UPHFP, most fellows have become public health leaders and have served as senior epidemiologists, project coordinators, and research associates for the MOH and partners. Fellows have obtained valuable knowledge and experience from completing their HIV and TB projects and use this knowledge in their postgraduate careers. Many fellows continue to support the HIV and TB response specifically for the MOH or partners.

## DISCUSSION

Since its inception, UPHFP has addressed multiple gaps in the Uganda health system, including in the HIV and TB response. Fellows have generated high-quality information products, which have been recognized nationally and internationally and have facilitated the adoption of their recommendations.^6^ As an integrated and adaptive program, UPHFP has also greatly improved Uganda's capacity to respond to public health emergencies and endemic diseases such as HIV and TB. Compared to implementing partners operating PEPFAR HIV/TB programs, UPHFP brings on board a unique approach to generating epidemiological evidence to inform decisions in the MOH. The TB operations research course offered to UPHFP fellows in 2018 focused on the important overlaps between TB and HIV and greatly contributed to the control of the TB and HIV dual epidemics in Uganda. Overall, MOH programs, including ACP and NTLP, have benefited not only from having all fellows complete at least 1 HIV- or TB-focused project but also from having a subset of fellows directly working with their sites to analyze data routinely and ensure that reports are generated promptly. Such efforts have, in turn, guided policies and program improvements.^13^^,^^14^ These data have been shared with government decision makers, international agencies, and nongovernmental organizations; most UPHFP findings have been published or are being prepared for publication. Other countries could benefit from similar initiatives in which public health professionals and trainees undertake high-impact projects with rapid turnaround to inform improvements in HIV and TB programs and policies.

UPHFP brings on board a unique approach to generating epidemiological evidence to inform decisions in the MOH.

## CONCLUSION AND PROGRAM IMPLICATIONS

During the past 6 years, UPHFP has significantly contributed to HIV and TB control in Uganda. Future steps that will ensure UPHFP HIV and TB projects are maximally informative and that results directly inform policy and program improvement include widening project identification and mentorship to include other key implementing partners and stakeholders. Other countries with similar programs could benefit from utilizing fellows to support HIV and TB epidemic control.

## Supplementary Material

GHSP-D-21-00574-supplement.pdf

## Notes

**Peer Reviewed**
