# Supplementary material for: Uganda Public Health Fellowship Program's Contributions to the National HIV and TB Programs, 2015–2020
Source: Glob Health Sci Pract. 2022 Apr 28;10(2):e2100574. doi: 10.9745/GHSP-D-21-00574 (PMC9053155; doi:10.9745/GHSP-D-21-00574)
Supplement: GHSP-D-21-00574-supplement.pdf [file GHSP-D-21-00574-supplement.pdf]

Supplement to: Ario AR, Bulage L, Wibabara Y, et al. Uganda Public Health Fellowship Program's contributions to the national HIV and TB programs, 2015–2020. *Glob Health Sci Pract*. 2022;10(2):e2100574. https://doi.org/10.9745/GHSP-D-21-00574

Supplement Table. Unpublished HIV and TB Projects Implemented by the Uganda Public Health Fellowship Program and Status of Publication, 2015–2020

S/No	Project Title	Year of Study or Investigation	Disease Type	Published
1	Improved facility performance in TB/HIV diagnosis and treatment after on-site mentoring mid-level providers	2015	TB/HIV	No
2	Twelve months retention in care among HIV clients on ART in Uganda as reported in HMIS	2015	HIV	No
3	Evaluation of the Integration of Isoniazid Preventive Therapy and Intensified TB Case Finding into comprehensive routine HIV care at Mildmay Uganda	2015	TB/HIV	No
4	Demographic and socio-economic risk factors for low CD4 count among adults testing HIV positive for the first time in Uganda: Case study of VCT-based surveillance at Mildmay Clinic	2016	HIV	No
5	Correlates of CD4 counts in HIV positive patients at Mildmay Uganda, 2011–2013	2016	HIV	No
6	Risk factors for HIV infection among persons aged 15–24 years: Case study of Voluntary Counseling and Testing-based surveillance at Mildmay Clinic, Kampala, Uganda – 2011–2013	2016	HIV	No
7	Prevalence and willingness of uptake of safe male circumcision among prisoners in Uganda	2016	HIV	No
8	Prophylactic treatment of HIV+ mothers and risk of infants' positivity	2016	HIV	No
9	Trends of weekly Short Message Service (SMS) reporting on Option B+ Indicators for Mildmay Uganda supported districts of Central Uganda, October 2016 to March 2017	2016	HIV	No
10	HIV Counseling and Testing as a best practice for preventing HIV transmission among Most at Risk Populations	2016	HIV	No
11	HIV viral load timeliness, test uptake and utilization for Achievement of viral load suppression using CQI approach	2016	HIV	No
11	Rapid Assessment of the Health Status of Refugees and HIV Services in Adjumani Refugee Settlement, Adjumani District	2016	HIV	No

12	Evaluation of the strategies used for referral, linkage to- and retention in HIV care for key populations at Mildmay Uganda and supported districts of Central Uganda, 2017	2016	HIV	No
13	Predictors of health-related quality of life among TB patients in Kampala	2016	TB	No
14	Assessment of Tuberculosis prevention, care and management in refugee settlements in West Nile, Uganda	2016	TB	No
15	Investigation of a suspected Tuberculosis outbreak in a School in Mukono District, Uganda, 2017	2016	TB	No
16	Retention of mothers on the Elimination of Mother to Child Transmission (eMTCT) of HIV program using Open Medical Record System (Open MRS) data	2017	HIV	No
17	Improving the quality of data recording for Option B+ weekly reporting and identification of approaches that can sustain the recording of good quality data	2017	HIV	No
18	A Continuous Quality Improvement project to improve on uptake, utilization, and timeliness of HIV Viral Load testing in two health facilities in Mbale District	2017	HIV	No
19	Rapid Assessment of HIV Service Delivery in Bidibidi Refugee settlement, Yumbe District, March 2017	2017	HIV	No
20	Outcomes of HIV status disclosure among adults attending HIV/AIDS care at Mildmay Uganda, 2008–2012	2017	HIV	No
21	Documentation of best practices on the implementation of HIV prevention among Most at Risk Populations (MARPs) by Mildmay Uganda	2017	HIV	No
22	Assessment of Integration of Isoniazid Preventive Therapy (IPT) and Intensified Tuberculosis Case Finding (ICF) into comprehensive Routine HIV Care at Mildmay Uganda	2017	HIV	No
23	Nutritional status and Outcomes of TB treatment in HIV co-infected TB patients in Mubende Regional Referral Hospital*	2017	TB/HIV	In peer review

24	Prevalence and outcomes of TB infection by HIV status in Kampala city, Uganda, 2016	2017	TB/HIV	No
25	A Comparison of Tuberculosis Treatment Outcomes among Children who are HIV positive and HIV negative in the Era of "Test and Treat", Uganda, 2016–2017	2017	TB/HIV	No
26	Bacteriologic Sputum Non-conversion among TB/HIV co-infected Smear Positive patients in Luwero District, Uganda*	2017	TB/HIV	In peer review
27	Trends, and spatial distribution of Presumptive Drug Resistant Tuberculosis in Uganda 2015–2017, a descriptive analysis from HMIS data	2017	TB	No
28	Spatial Distribution and Temporal Trends of Tuberculosis Notifications in Uganda: A Five-Year Retrospective Analysis (2013–2017)	2017	TB	No
29	Predictors of Treatment Outcome of Tuberculosis patients aged ≥8 years accessing Treatment under Directly Observed Treatment Strategy in Lira Regional Referral Hospital, Uganda*	2017	TB	In peer review
30	Assessment and characterization of tuberculosis treatment outcomes among adolescents and young adults in Jinja Regional Referral Hospital, Eastern Uganda*	2017	TB	In peer review
31	Improving Tuberculosis Reporting in Lira District Using Continuous Quality Improvement Science, June 2018	2017	TB	No
32	Nutritional Status and Tuberculosis Mortality in Kyangwali Refugee Settlement, Hoima District: 2017*	2017	TB	In peer review
33	Deaths in Tuberculosis Patients compared with TB/HIV co-infected Patients above 14 years in Lira Regional Referral Hospital, Uganda 2012 to 2017*	2017	TB/HIV	In peer review
34	Comparison of Tuberculosis treatment outcomes in HIV positive and HIV negative children aged 0–14 years in Uganda, 2016–2017*	2017	TB	In peer review
35	Survival of TB/HIV co-infected patients in Kampala and associated factors*	2017	TB/HIV	In peer review
36	Understanding the demographic, health and social characteristics of HIV positive mothers who get Lost to Follow Up from the Prevention of Mother to Child Transmission program	2017	HIV	No

37	Improving the screening, management and reporting of early cryptococcal disease among HIV positive patients at Nkozi Hospital, in Mpigi district, Uganda- 2018	2017	HIV	No
38	Factors Associated with Treatment Outcomes among Pediatric Tuberculosis Patients at Jinja Regional Referral Hospital, Uganda, 2016–2017*	2017	TB	In peer review
39	Addressing Low Proportions of Repeat HIV Testing in Women in Maternity in Kayunga Hospital, Uganda, 2018: A Quality Improvement Project	2017	HIV	No
40	Prevalence and Associated risk factors of HIV infection among women prisoners in Uganda Prisons Services	2017	HIV	No
41	Assessing conversion to HIV-positivity among babies on Option B+ Program for Prevention of Mother to Child Transmission of HIV: Kampala and Wakiso Districts, 2012–2018	2017	HIV	No
42	Predictors for HIV and syphilis co-infection among mothers seeking antenatal care at Tororo Hospital, 2015–2016	2017	HIV	No
43	Burden and correlates of neuro-psychiatric disorders among HIV positive prisoners in Uganda, 2015	2017	HIV	No
44	High burden of HIV, syphilis, and schistosomiasis and low access to health services among fisher folks in the Lake Kyoga Fishing community, Uganda, 2013	2017	HIV	In peer review
45	Development of tools and Standard Operating Procedures for offering Pre-Exposure Prophylaxis to HIV negative populations at risk of acquiring HIV	2017	HIV	No
46	Improving quality of reporting of Option B+ indicators using continuous quality improvement approaches in Entebbe Hospital	2017	HIV	No
47	Predictors for treatment outcomes among Multidrug-resistant tuberculosis patients attending Lira and Arua Regional Referral Hospitals, Uganda: 2012–2017*	2017	TB	In peer review
48	Predictors of loss to follow up among TB patients – a comparison of the 2012–2013 and 2015–2016 Cohorts*	2017	TB	In peer review

49	Investigation of an Outbreak of Extra-pulmonary TB in Napak	2017	TB	No
50	Completion rates for a 6-month course of IPT among PLHIV started on IPT and predictors of non-completion in the Soroti Region, 2015 to 2017*	2018	TB/HIV	In peer review
51	Analysis of Viral Load Suppression Trends and predictors of non-suppression among young adults aged 15 to 24 years in Uganda from 2014 to 2018	2018	HIV	No
52	Burden of HIV, and risk factors for infection among men who have sex with men in Ugandan prisons, 2013–2014	2018	HIV	In peer review
53	Prevalence and distribution of Tuberculosis and HIV Co-infection among Refugees in Rhino Camp Refugee Settlement, Arua District, 2015–2017	2018	TB/HIV	No
54	Predictors for Mortality among Multidrug Resistant Tuberculosis and HIV co-infected patients in Uganda, 2013–2016*	2018	TB/HIV	In peer review
55	Trends and distribution of presumptive Tuberculosis among newly diagnosed HIV patients in Uganda 2013–2017*	2018	TB/HIV	In peer review
56	Trends and distribution of presumptive Tuberculosis among newly diagnosed HIV patients in Uganda 2013–2017	2018	TB/HIV	No
57	Incidence and risk factors of active tuberculosis among HIV positive prisoners on ART in Uganda 2013–2017	2018	TB/HIV	In peer review
58	Risk Factors for Active Tuberculosis among HIV-positive Prisoners on ART at Murchison Bay Hospital in Luzira Maximum Security Prison, Uganda; 2013–2017	2018	TB/HIV	No
59	Assessment and Characterization of Extra-Pulmonary Tuberculosis in Uganda, 2014–2017	2018	TB	No
60	The Burden and Treatment outcomes of Pediatric Tuberculosis in Eastern Uganda, 2013–2017*	2018	TB	In peer review
61	Comparing Characteristics and Treatment Outcomes of Rifampicin Mono-resistant Tuberculosis and Multi Drug- resistant Tuberculosis Cases in Uganda*	2018	TB	In peer review

62	Assessment and Characterization of Extra-Pulmonary TB in Uganda Between 2014 And 2017*	2018	TB	In peer review
63	Turnaround Time of Xpert MTB Assay in Routine Clinical Practice and Time to Initiation on Treatment for patients with Rifampicin Resistant Xpert Results: A Case Study of Iganga Hospital, Uganda*	2018	TB	In peer review
64	Investigation of a tuberculosis outbreak in a national referral psychiatric hospital in Kampala District, Uganda	2018	TB	No
65	Sero-prevalence and Factors Associated with Hepatitis B Virus Infection among Prisoners and Prisons Staff in Uganda Prisons, 2013/2014	2018	HIV	In peer review
66	Factors Associated with Treatment Outcomes among Multidrug-Resistant Tuberculosis Patients in Uganda: A Comparative Analysis of Cohorts 2013 to 2015*	2018	TB	In peer review
67	Prevalence and distribution of TB/HIV co-infection among refugees in Rhino Camp refugee settlement, Arua District*	2018	TB	In peer review
68	Factors Associated with Treatment Outcomes among Multidrug-Resistant Tuberculosis Patients in Uganda: A Comparative Analysis of Cohorts 2013 to 2015*	2018	TB	In peer review
69	Surveillance data analysis on trends and distribution of TB disease in Moroto region, 2010–2017	2018	TB	No
70	Comparison of Early Infant Diagnosis data using District Health Information Software2, Central Public Health Laboratory databases, and Health Facility data sources to assess performance of EID Program in Uganda, 2015–2018	2018	HIV	No
71	HIV among pregnant mothers attending antenatal care at Tororo Hospital	2018	HIV	No
72	Factors associated with HIV among Adolescent and Young Adult Female Sex Workers in Kampala	2018	HIV	No
73	Sexual violence, HIV infection and STDs among male prisoners in Uganda	2018	HIV	No
74	Mental Health among HIV+ Prisoners in Uganda	2018	HIV	No
75	Characterizing Non-Occupational PEP in Uganda 2014–2018 - Implications for PrEP Programs	2018	HIV	No

76	Predictors for low viral load suppression among adult HIV patients in Uganda	2018	HIV	No
77	Prevalence and Distribution of HIV among people that experience childhood abuse	2019	HIV	No
78	Factors influencing Turnaround Time of GeneXpert Test Results at Lower Level Health Facilities, Kampala City, Uganda, January-December 2018	2019	TB	In peer review
79	Comparison of maternal and perinatal outcomes among HIV positive and HIV negative women in SMGL programs in Uganda, 2015–2016	2019	HIV	No
80	Progress towards 90-90-90 for prisoners in prisons providing direct HIV services versus services via referral, Uganda: A cascade analysis for 2017–2018.	2019	HIV	In peer review
81	Exploring the Use of High Viral Load to Identify Advanced HIV Disease	2019	HIV	No
82	Assessment of the of layering of HIV interventions given to adolescent girls and Young Women in the DREAMS project in Uganda	2019	HIV	No
83	Improving Recency Testing in Rwenzori Region Using Continuous Quality Improvement Approaches, Uganda 2020	2019	HIV	No
84	Factors associated with late 1st DNA PCR test among HIV exposed infants in Fort Portal Region, Uganda, July-December 2019	2019	HIV	No
85	HIV Care Cascade among Key Populations in Uganda: A retrospective Cohort study	2019	HIV	No
86	Intimate Partner Violence associated with Assisted Partner Notification in HIV Clinics in Uganda	2019	HIV	No
87	HIV/TB Co-Infection and associated factors among new Prisoners on admission to Luzira Prisons, 2014–2018	2019	TB/HIV	No
88	Investigation of TB diagnostic discrepancy in selected prisons of Uganda, 2020	2019	TB	No
89	Contraceptive use among adolescent girls and young women engaged in commercial sex work, Kampala District, 2013–2018	2019	TB	No
90	MDR TB Investigation in Nakapiripirit District	2019	TB	No
91	Evaluating increased reports of liver toxicity following Isoniazid prophylaxis in Kampala	2019	TB	No

92	Validation of a computer-based HIV risk scoring tool for predicting HIV positivity among key populations in Uganda	2019	HIV	No
93	Correlates of early retention among patients living with HIV newly initiated on treatment in Wakiso and Kampala Districts 2017–2018	2019	HIV	No
94	Investigation of a suspected Leprosy outbreak in Lira District, Uganda, July 2018 to February, 2019	2019	Leprosy	No
95	Assessment of Viral Load Suppression among ART patients as a predictor of Advanced HIV disease	2020	HIV	No
96	Determinants of age at sexual debut among adolescents in Uganda	2020	HIV	No
97	Determinants of access and utilization of maternal and child health services in saving mothers giving live programs in Uganda: A comparison of maternal & perinatal outcomes among HIV + women and HIV – women	2020	HIV	No
98	Comparison of TB data quality reported in DHIS2 and NTLP	2020	TB	No
99	Predictors of and reasons for late 1st DNA PCR testing among HIV-exposed infants	2020	HIV	No
100	Uptake of VMMC in Uganda, A comparison of surgical and device methods	2020	HIV	No
101	Trends of ANC Attendance and HIV Positivity among women and Exposed Infants in Uganda, 2014–2018	2020	HIV	No
102	A Descriptive analysis of surveillance data on the side effects of DTG in Uganda, 2017–2019	2020	HIV	No
103	The impact of SLMTA in implementing quality-assured laboratory services in Uganda	2020	HIV	No
104	Factors affecting Turn Around Time of Sputum Test Results at lower level health Facilities in Kampala	2020	HIV	No
105	Use of Recency data for public health approach to HIV epidemic control efforts	2020	HIV	No
106	Risk and protective factors for violence at childhood and the HIV Cascade among children 15–24 years in Uganda	2020	HIV	No
107	An HIV Care cascade analysis among inmates in facilities Uganda Jan 2016–2017	2020	HIV	No

108	Tuberculosis Preventive Treatment Scale-Up Among Antiretroviral Therapy Patients in Hoima district, Uganda 2021	2020	TB/HIV	No
109	Effect of community differentiated service delivery models on retention and viral suppression among stable HIV clients in Kampala and Wakiso districts, Uganda	2020	HIV	No
110	Assessing retention and predictors to completion of EID among HIV exposed infants in Rwenzori Region in Uganda	2020	HIV	No

*TB Operations Research Project

Supplement to: Ario AR, Bulage L, Wibabara Y, et al. Uganda Public Health Fellowship Program's contributions to the national HIV and TB programs, 2015–2020. *Glob Health Sci Pract*. 2022;10(2):e2100574. https://doi.org/10.9745/GHSP-D-21-00574
